# Speciation Progress: A Case Study on the Bushcricket *Poecilimon veluchianus*

**DOI:** 10.1371/journal.pone.0139494

**Published:** 2015-10-05

**Authors:** Lucienne Eweleit, Klaus Reinhold, Jan Sauer

**Affiliations:** 1 Department of Evolutionary Biology, Bielefeld University, Bielefeld, Germany; 2 Department of Chemical Ecology, Bielefeld University, Bielefeld, Germany; University of Massachusetts, UNITED STATES

## Abstract

Different mechanisms such as selection or genetic drift permitted e.g. by geographical isolation can lead to differentiation of populations and could cause subsequent speciation. The two subspecies of *Poecilimon veluchianus*, a bushcricket endemic to central Greece, show a parapatric distribution and are partially reproductively isolated. Therefore, *P*. *veluchianus* is suitable to investigate an ongoing speciation process. We based our analysis on sequences of the internal transcribed spacer (ITS) and the mitochondrial control region (CR). The population genetic analysis based on the nuclear marker ITS revealed a barrier to gene flow within the range of *Poecilimon veluchianus*, which corresponds well to the described subspecies. In contrast to the results based on the nuclear ITS marker, the mitochondrial CR marker does not clearly support the separation into two subspecies with restricted gene flow and a clear contact zone. Furthermore, we could identify isolation by distance (IBD) as one important mechanism responsible for the observed genetic structure (based on the ITS marker). The population genetic analysis based on the nuclear marker ITS also suggests the existence of hybrids in the wild. Furthermore, the simultaneous lack of strong prezygotic barriers and the presence of postzygotic mating barriers, observed in previous laboratory experiments, suggest that a secondary contact after an allopatric phase is more likely than parapatric speciation.

## Introduction

Speciation is still a hot topic in evolutionary biology, and different mechanisms and geographic modes of speciation have been proposed to cause this event [1–4]. Selection and genetic drift, permitted by e.g. geographical isolation may lead to differentiation of populations and could cause subsequent speciation [5]. The observed genetic variation is often used to reconstruct current or past patterns of gene flow in a species [6]. Historical geographic processes such as population division, range expansion and long distance colonization and the relationships between them, are expected to produce distinct pattern in allele distributions [7]. Hence, it is reasonable to infer those processes from pattern of genetic variation [8].

**Fig 1:**
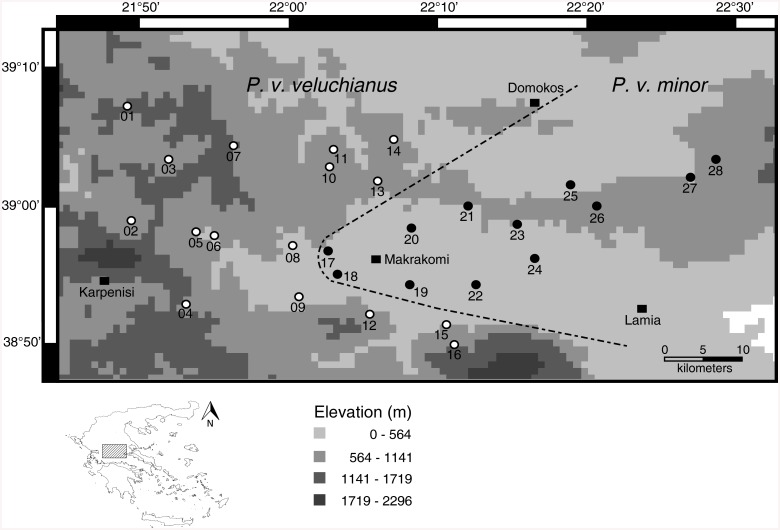
Sampling locations in Central Greece for *P*. *v*. *veluchianus* (left side, open circles) and *P*. *v*. *minor* (right side, filled circles) with hypothesized contact zone (dashed line). The Map was prepared in DIVA-GIS version 7.5.0.0 using an elevation shapefile freely available at https://research.cip.cgiar.org/gis/.

We chose *Poecilimon veluchianus*, a flightless bushcricket, which is endemic to central Greece, as a promising system to investigate forces that could drive speciation. The two subspecies, *P*. *v*. *veluchianus* and *P*. *v*. *minor* [9], are described based on significant differences in body size and behavioral traits like sperm transfer rate and signaling duration per day [9,10]. Size differences were found between several populations, and were confirmed for almost the entire distribution range [11], whereas the behavioral differences were observed in a one population comparison close to Vitoli in central Greece (sample sites 08 and 17 in Fig 1) [10]. The two subspecies are parapatrically distributed with a V-shaped contact zone (Fig 1) within central Greece [9,11].

As elevation might influence the distribution of the two subspecies [11], the Iti Mountain could be a geographical barrier to gene flow. In the northern part of the distribution, the contact zone between the two subspecies is difficult to determine, due to agricultural areas in which *P*. *veluchianus* rarely occurs. As speciation might occur over large time scales, it is important to consider past fragmentations that might have served as potential barriers to gene flow. During ice ages in the Quaternary, some 2.4 million years ago, central Greece was not fragmented [12] and might have served as a refuge for several species. This suggests that the geographical range of the species occurring in this area should have been rather constant.

However, previous laboratory experiments have shown that, despite the females do not distinguish between songs of the two subspecies [9],the mating success of both subspecies is impeded by partial postzygotic isolation, which reduces the amount of sperm transfer and influences the fertility of the F1 generation [10]. Cross-mating experiments have shown that females of the F1-hybrid-generation were mostly fertile, but males of one cross of the F1-hybrid-generation had lower sperm numbers and were mostly sterile. Thus, there seems to be evidence for the presence of postzygotic isolation mechanisms but missing premating isolation mechanisms. Besides, these two subspecies also differ in several phenotypic traits such as body size, the timing of male signaling and the duration necessary for sperm transfer [13,14], which indicates that speciation might be an ongoing process.

To test for possible genetic differences between the proposed subspecies [9,10,13], we investigated the population structure of *P*. *veluchianus* using sequences of the mitochondrial control region (CR marker) and the internal transcribed spacers (ITS marker 1 and 2). Due to the lack of premating barriers [9], hybridization might occur in the area of the contact zone, which should enhance gene flow between the subspecies in the contact zone in comparison to more distantly located sites [15]. To test the hypothesis that hybridization of both subspecies happens in the contact zone, we analyzed whether higher amounts of shared haplotypes occur in the contact zone in comparison to more distantly related sampling sites. To examine whether isolation by distance (IBD) might have shaped the population structure of both subspecies and which possible mechanisms might best explain the distribution pattern of both subspecies, we performed a distance-based redundancy analysis (dbRDA).

Our investigations revealed one main barrier rather similar to the contact zone formerly hypothesized due to phenotypic differences. IBD has great influence on the genetic structure observed, but secondary contact seems to be rather likely than ongoing speciation in parapatry.

## Materials and Methods

### Sampling

*P*. *veluchianus* is a flightless bushcricket with two parapatrically distributed subspecies that are endemic to central Greece. In three different years, 2010–2012, we collected in total 283 individuals at 28 sites. *P*. *v*. *veluchianus* was collected at 16 sites and *P*. *v*. *minor* at 12 sites (Fig 1). The material was immediately stored in absolute ethanol. We proposed to collect comparable numbers of males and females for every sample site. No specific permission was required for this study as this bushcricket species is not listed in the Red List of the International Union for Conservation of Nature (IUCN), and the collection of non-vertebrate species is permitted in Greece. All Maps were prepared in DIVA-GIS version 7.5.0.0 [16] using an elevation shapefile freely available at https://research.cip.cgiar.org/gis/.

### Sample preparation

DNA was extracted from all individuals by the chloroform-isoamyl approach [17].

The mitochondrial CR marker was amplified using primer SR-J14610 (ATAATAGGGTATCTAATCCTAGT) and a modified TI-N18 primer (CTCTATCAARRTAAYCCTTT) [18]. Sequences of internal transcribed spacer (ITS) 1 to 2 including the intermediate 5.8S rRNA were amplified using primers (TAGAGGAAGTAAAAGTCG and GCTTAAATTCAGCGG) described in Weekers *et al*.(2001) [19]. These markers are expected to have a high resolution and be useful to distinguish between species, subspecies and even populations [20, 11].

To amplify the mitochondrial CR marker the following PCR protocol was used: 1.2 μl genomic DNA, 0.1 μl innuTaq Hot-A DNA Polymerase (Analytic Jena), 2.5 μl Hot Start Buffer complete, 1.2 μl of a 10 μM solution of each primer, 0.6 μl of a 2 mM dNTP-Mix and ddH_2_O added to a final volume of 30 μl. According to Zhao *et al*. (2011) [21], the PCR conditions included an initial denaturation of 2 min at 92°C, followed by 35 cycles with 92°C for 20 s, 52°C for 30 s, and 60°C for 3 min. After the final elongation step of 7 min at 72°C the samples were cooled down to 10°C and stored at -20°C until sequencing.

To amplify fragments of the ITS marker the following PCR protocol was used: 0.8 μl genomic DNA, 0.07 μl innuTaq Hot-A DNA Polymerase (Analytic Jena), 1.7 μl Hot Start Buffer complete, 0.8 μl of a 10 μM solution of each primer, 0.4 μl of a 2 mM dNTP-Mix and ddH_2_O added to a final volume of 20 μl. According to Weekers *et al*. (2001) [19] the PCR conditions included an initial denaturation of 4 min at 95°C, followed by 30 cycles with 95°C for 1 min, 52°C for 1.5 min, and 72°C for 2 min. After the final elongation of 10 min at 72°C the samples were cooled down to 10°C and stored at -20°C until sequencing.

After purifying, following the ExoSAP-IT PCR clean-up protocol (USB Corporation), PCR products were directly cycle-sequenced using the amplification primers and then analyzed on an ABI 3730 capillary sequencer (Applied Biosystems) at the sequencing facility of the CeBiTec (Bioinformatics Resource Facility) of Bielefeld University.

In the resulting analysis datasets for both markers (ITS and CR) contained sequences of the same 283 individuals (*P*. *v*. *veluchianus* = 164 sequences, *P*. *v*. *minor* = 119 sequences). The assembly of forward and reverse sequences was performed in MEGA 5.2 [22]. The sequences of both datasets were aligned with the implemented ClustalW algorithm [23] in MEGA 5.2 and afterwards the alignment was improved by hand (alignments available as S1 File for the CR marker and S2 File for the ITS marker). Raw-Sequences analyzed in this article have been deposited in gene bank [GenBank: KP941779—KP942344].

For both datasets we performed a model test to find the best model of sequence evolution for the following analyses, implemented in MEGA 5.2. Gaps were excluded for this analysis. Models were chosen based on the Akaike information criterion (AIC).

### Genetic structure and subspecies delamination

The genetic structure and diversity of the datasets were investigated with the program DNAsp5 [24]. At first the sequential datasets were transformed to haplotype datasets with frequencies per haplotype per sample site. In addition, for a better understanding of the underlying diversity, average number of pairwise differences for pairs of all sample sites were calculated using the program Arlequin 3.5.1.2 [25].

To find out whether the subspecies are supported by the Fst-values we used the software Barrier v. 2.2 [26] and its implemented Monmonier’s maximum difference algorithm. To calculate the robustness of the computed barriers the raw data were bootstrapped 100 times to calculate 100 Fst-Matrices for every pair of sample sites by the Program R version 3.0.3 [27] using the package PopGenome version 2.0.6 [28].

To determine the percentage of genetic variation explained by the affiliation into subspecies a distance based redundancy analysis (dbRDA) [29,30] was performed by using DistLM v.5 [31]. This regression analysis was based on a p-distance-matrix calculated for each pair of individuals by MEGA 5.2, as the independent variable. The Dependent variable was the arrangement of the individuals to the subspecies calculated by Barrier. We performed the dbRDA with the partitioning of the individuals to the hypothesized subspecies and the new arrangement of sample sites to subspecies via the first barrier calculated by Barrier for the ITS marker. Significances of the dbRDA were established with 9999 permutations in DISTLM v. 5.

To further investigate the genetic structure of the studied species and subspecies we performed an AMOVA. The AMOVA was calculated by using the program Arlequin 3.5.1.2 based on the haplotype information, provided by DNAsp5 and the affiliation of the sample sites to the both subspecies and additionally the new affiliation into the subspecies as calculated by Barrier for the ITS marker. Significance test was performed with 1023 permutations.

### Mechanisms of separation

We tested for IBD by performing a dbRDA on three different arrangements (overall dataset without any partitioning, affiliation to hypothesized subspecies as covariate, and affiliation to newly calculated subspecies as covariate) of the p-distance-matrix of the individuals and the associated geographical coordinates as variables. We also tested both sexes separately on the whole dataset without any partitioning to test for sex biased dispersal. Again, significances of the dbRDA were established with 9999 permutations in DISTLM v. 5.

To test whether the analyzed sequences show departure from neutral evolution, we calculated Tajimas D and Fu’s and Li’s F* and D* as implemented in DNAsp5. We performed both tests as Fu’s and Li’s F* and D* are supposed to be more sensitive to population expansions and genetic hitchhiking than Tajimas D [32]. Both tests were performed with DNAsp5 and the arrangement of sample sites into the hypothesized subspecies, respectively in accordance to the calculated barrier.

## Results

### Modeltest

The aligned sequences of the CR dataset contained 794 bp and the ITS dataset consisted of 706 bp. For the CR dataset the model test resulted in GTR+G+I (G = 0.43, I = 0.54, R = 2.80) with the lowest AIC value. This model was used for calculation of the p-distances in MEGA 5.2. As this model is not provided in Arlequin 3.5.1.2, for this program we chose the model with the next best AIC value of the selectable models, Tamura-Nei (G = 0.184).

The model with the lowest AIC value for the ITS dataset was T92+G+I (G = 0.28, I = 0.89, R = 1.31) in the model test. This model was used for calculation of the p-distances in MEGA 5.2. For Arlequin 3.5.1.2 the model Kimura2P+G (G = 0.05, R = 1. 26) had the lowest AIC value and was therefore used in the Arelquin analyses.

### Genetic structure

**Fig 2:**
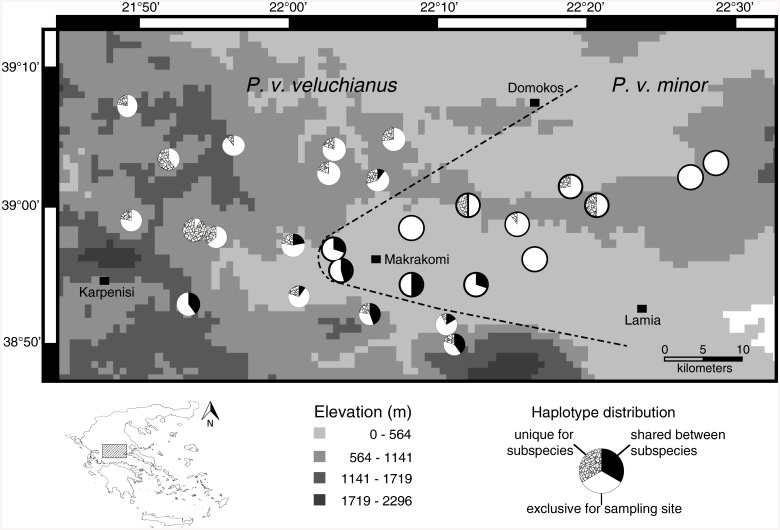
Distribution of shared and exclusive haplotypes of the CR marker regarding to their frequencies, with hypothesized contact zone (dashed line). The Map was prepared in DIVA-GIS version 7.5.0.0 using an elevation shapefile freely available at https://research.cip.cgiar.org/gis/.

**Fig 3:**
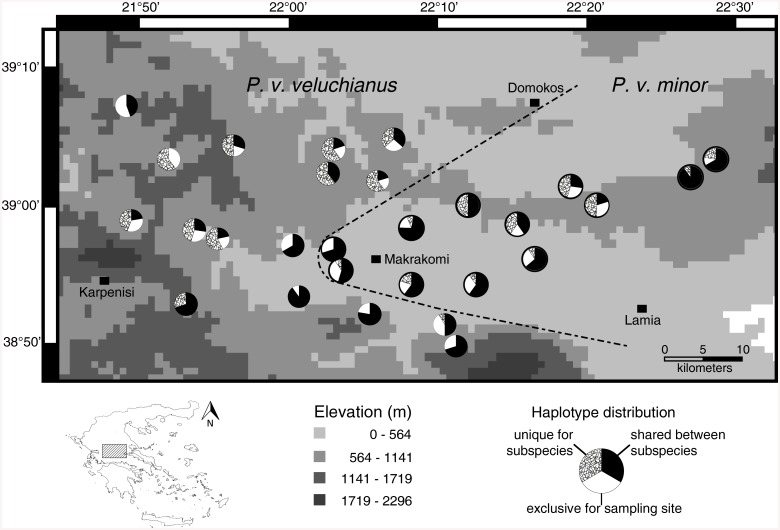
Distribution of shared and exclusive haplotypes of the ITS marker regarding to their frequencies, with hypothesized contact zone (dashed line). The Map was prepared in DIVA-GIS version 7.5.0.0 using an elevation shapefile freely available at https://research.cip.cgiar.org/gis/.

The mitochondrial CR marker as well as the nuclear ITS marker showed high genetic variation (Table 1). For both markers this high genetic variation is due to a relatively low number of variable sites. The haplotype diversity for both subspecies was high and did not differ largely between used markers and subspecies (Pvv: CR mean ± SD = 0.989 ± 0.003, ITS = 0.973 ± 0.005; Pvm: CR = 0.988 ± 0.003, ITS = 0.979 ± 0.004), but as expected the used nuclear marker (ITS) displayed a lower genetic variation. A characteristic of the studied species is a very high number of exclusive haplotypes occurring only at one of the different sampling sites which is most obvious in the mitochondrial CR marker and holds true for both subspecies (see Fig 2 and S3 File). In contrast, the ITS marker showed much lower numbers of sample site specific haplotypes as the CR marker (Fig 3 and S4 File). Despite the high haplotype diversity in both datasets, the p-distances between and within the sample sites are low (S1 and S2 Figs).

**Table 1:** Genetic structure and diversity, according to the hypothesized subspecies.

dataset		n	haplotypes	haplotypes shared between subspecies	single haplotypes	haplotype diversity	± SD	invariable sites [bp]	variable sites [bp]	total no. of mutations [bp]	parsimony informative mutations [bp]
**Pvv**	**CR**	164	98	-	65	0.989	0.003	612	182	212	147
	**ITS**	164	78	-	39	0.973	0.005	679	27	29	18
**Pvm**	**CR**	119	75	-	54	0.988	0.003	635	159	189	136
	**ITS**	119	57	-	24	0.979	0.004	682	24	25	13
**CR**		283	168	5	119	0.993	0.001	591	203	250	161
**ITS**		283	111	24	62	0.982	0.002	665	40	41	21

### Subspecies delimitation

**Fig 4:**
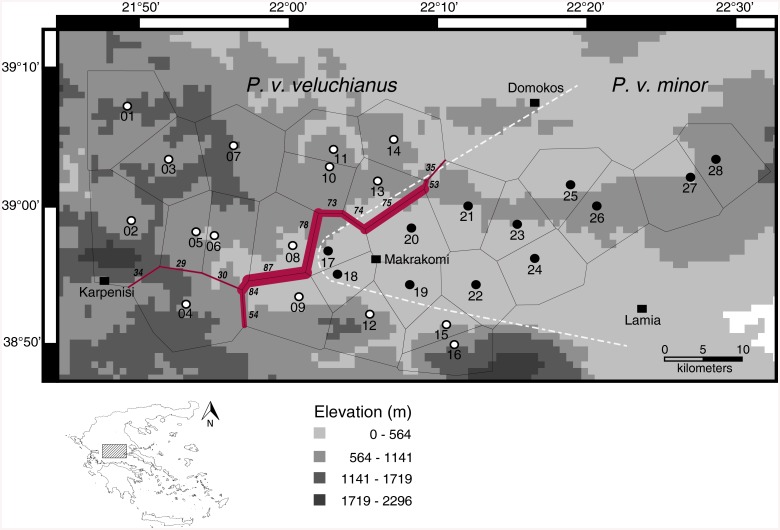
Calculation of new contact zone, based on the ITS marker. Sample sites in central Greece with hypothesized contact zone (white dashed line). The first calculated barrier for the ITS marker is shown in red, italic numbers are the bootstrapping values for every decision made along the barrier. Voronoï tessellation by the software Barrier is shown as thin black lines around all sample sites. The Map was prepared in DIVA-GIS version 7.5.0.0 using an elevation shapefile freely available at https://research.cip.cgiar.org/gis/.

To ascertain whether the assignment of the individuals to subspecies is supported by the Fst-values for both markers, the first four barriers to gene flow were calculated using the software Barrier v. 2.2. The first calculated barrier for the ITS marker with their bootstrapping values for every decision is shown in Fig 4. All barriers calculated afterwards received only bootstrap support values lower than 30 and are therefore not shown as they seem to have low explanatory value to the population structure. For the CR marker also only the first reconstructed barrier received high bootstrap support values. This barrier isolates two sampling sites (27 and 28) in the eastern most range of *P*. *v*. *minor* from all remaining sampling sites (data not shown). This reconstructed barrier is not corresponding to an obvious geographic barrier and is not adding further information on the question about the subspecies or the genetic pattern concerning the two subspecies. Moreover, the mitochondrial CR marker did not support two different subspecies with restricted gene flow.

The barriers to gene flow determined are not exactly in accordance to the hypothesized contact zone, but barrier 1 calculated for the ITS marker supports it almost as proposed by Eweleit & Reinhold (2014) [11]. Just the affiliation of four to five sample sites in the south, which were originally attributed to the subspecies *P*. *v*. *veluchianus*, was rather unexpected. The calculated barrier indicates that these *P*. *v*. *veluchianus* populations are more similar to the adjacent *P*. *v*. *minor* populations and hence, did not correspond to the proposed contact zone. Many of the decisions made by Barrier are supported by bootstrap values higher than 70%. In the north-east the contact zone between sample site 14 and 21 is only supported by a bootstrap value of 35. In the south two possible ways for the continuing barrier to gene flow were calculated. The barrier between sample site 04 and 09 is supported by a bootstrap value of 54. The other possible way of the contact zone, including sample site 04 to *P*. *v*. *minor*, is supported by bootstrap values lower than 25. Low bootstrap values may suggest unsampled locations [33], the presence of hybrids or a certain amount of gene flow in these areas [34]. Between sample site 14 and 21 a missing sample site seems to be plausible explanation, as in this region, due to agricultural use, the finding of sample locations was difficult.

**Fig 5:**
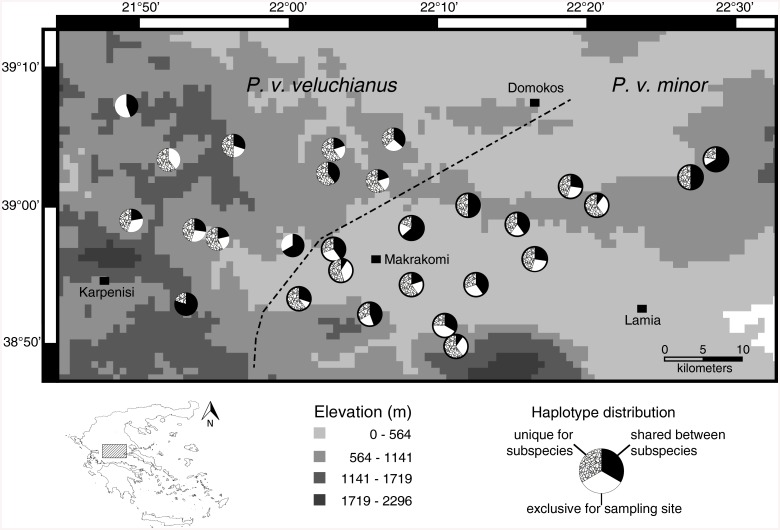
Distribution of shared and exclusive haplotypes of the ITS marker regarding to their frequencies, with new contact zone (dashed line). The Map was prepared in DIVA-GIS version 7.5.0.0 using an elevation shapefile freely available at https://research.cip.cgiar.org/gis/.

Based on the bootstrap support values we assume the most possible contact zone as shown in Fig 5. See S5 File and Fig 4 for numbers of site specific haplotypes according to the new contact zone.

For further analysis we will focus on the ITS marker, to focus on the contact zone between the two subspecies calculated by barrier. The CR marker is not diagnostic in this respect, as it shows too much variability to resolve on the subspecies level. The Genetic structure and diversity for the ITS marker, according to the new subspecies is shown in Table 2.

**Table 2:** Genetic structure and diversity for the ITS marker, according to the new subspecies.

dataset	n	haplotypes	haplotypes shared between subspecies	single haplotypes	haplotype diversity	± SD	invariable sites [bp]	variable sites [bp]	total no. of mutations [bp]	parsimony informative mutations [bp]
**Pvv**	123	58	-	35	0.960	0.009	680	26	27	15
**Pvm**	160	68	-	41	0.976	0.004	680	26	27	15

The result of the dbRDA supports the calculated barrier (Table 3). The affiliation of individuals to the hypothesized subspecies was significant for the ITS marker (pseudo F = 28.6, permutation P < 0.001 and R^2^ = 0.092). The partitioning of individuals into the corresponding subspecies with respect to the newly calculated contact zone (barrier 1) for the ITS marker was also significant and is adding some more explanation of the genetic variation (pseudo F = 54.355, permutation P < 0.001 and R^2^ = 0.162).

**Table 3:** Results of dbRDA for the calculated regions/partitions for the ITS marker.

	pseudo F	permutation P	R^2^
hypothesized subspecies	28.6	< 0.001	0.092
new subspecies (barrier 1)	54.36	< 0.001	0.162
sex	1.17	> 0.3	0.004
**IBD**			
overall dataset	25.91	< 0.001	0.156
Pvv	10.46	< 0.001	0.149
Pvm	11.5	< 0.001	0.128
Males	7.9	< 0.001	0.103
Females	16.79	< 0.001	0.193

The amount of explained variation of the genetic pattern in the ITS marker, analyzed by an AMOVA, is different for the diverse genetic levels (individuals, sample sites and subspecies; S6 and S7 Files). When using the partitioning of the sample sites into the subspecies along the originally hypothesized contact zone, 10.69% (p < 0.0001) of the variation was explained by subspecies. Another 17.21% (p < 0.0001) of the variation was explained among the sample sites within the subspecies and 72.1% (p < 0.001) of the variation was explained within the sample sites. By the affiliation of the sample sites into the subspecies along the calculated barrier 1 for the ITS marker 18.47% (p < 0.0001) of the variation is explained by the subspecies. 12.3% (p < 0.0001) of the variation is explained among the sample sites within the subspecies and 69.23% (p < 0.0001) of the variation is explained within the sample sites. The new partitioning of the sample sites to subspecies increased the variation explained by subspecies for the ITS marker remarkably (by more than 70%).

Compared to the hypothesized subspecies, the analyses presented here revealed a slightly different contact zone for the two subspecies. Therefore, we further focus on the newly identified contact zone calculated by the software Barrier and the dbRDA. Hence, we are concentrating on the arrangement of sample sites along the newly established subspecies; results based on the previously hypothesized subspecies are explicitly mentioned.

### Mechanisms of separation

The test for IBD as a force for separation revealed 15.62% of the genetic variation was explained (pseudo F = 25.91, permutation P < 0.001 and R^2^ = 0.156) by using the geographical coordinates as variable. Within the two subspecies the influence of IBD is almost the same as for the overall dataset (Pvv: pseudo F = 10.46, permutation P < 0.001 and R^2^ = 0.149; Pvm: pseudo F = 11.5, permutation P < 0.001 and R^2^ = 0.128).

To test for the influence of sex on the genetic pattern we also performed the dbRDA based on the p-distances and the sex of each individual (Table 3). The result of this analysis was not significant (pseudo F = 1.17, permutation P > 0.3 and R^2^ = 0.004), indicating that sex has no strong influence on the observed genetic structure. Furthermore, this contradicts the expectation that females, as the songless sex, should be more actively walking around and hence could have a higher impact on the population structure.

To investigate whether the sequences show signs of non-random effects or evolve neutrally, we calculated Tajima’s D and the Fu’s and Li’s F* and D* (Table 4). For the ITS dataset the Tajima D values were not significant, but Fu’s and Li’s F* and D* were significant for the whole dataset (F* = -3.303, p < 0.02; D* = -4.189, p < 0.02). We found the same for the arrangement of the sample sites for the original hypothesized subspecies *P*. *v*. *minor* (F* = -2.351, p < 0.05; D* = -2.889, p < 0.05) and also for the new arrangement of sample sites into the subspecies *P*. *v*. *minor* along barrier 1 (F* = -2.498, p < 0.05; D* = -2.248, p < 0.05). Hence, the individuals in these areas seem to be under some sort of selection and the DNA- sequences do not evolve randomly. Population growth or population subdivision may also be a possible explanation as these genetic signatures are hardly to distinguish from selection effects [35].

**Table 4:** Summary of Tajima D test and Fu’s and Li’s F* and D* test of the ITS dataset.

	Tajimas D	P	Fu’s and Li’s F*	P	Fu’s and Li’s D*	P
overall	-0.899	> 0.1	**-3.303**	**< 0.02**	**-4.189**	**< 0.02**
original Pvv	-0.333	> 0.1	-1.491	> 0.10	-1.864	> 0.10
original Pvm	-0.512	> 0.1	**-2.351**	**< 0.05**	**-2.889**	**< 0.05**
new Pvv	-0.552	> 0.1	-1.894	> 0.05	-2.248	> 0.05
new Pvm	-0.528	> 0.1	**-2.498**	**< 0.05**	**-3.134**	**< 0.05**

## Discussion

The population genetic analysis based on the nuclear marker ITS revealed one main barrier to gene flow, resulting in partially reproductive isolation [10], within the range of *Poecilimon veluchianus*. This contact zone is diagonally extending from the north-east of central Greece to the south-west (Fig 5), through the Iti Mountains and separates the two subspecies *P*. *v*. *veluchianus* and *P*. *v*. *minor*, which had been described earlier based on significant differences in body size and behavioral traits [9]. The reconstructed barrier to gene flow corresponds well to a hypothesized contact zone/hybrid zone between subspecies which was postulated by Eweleit & Reinhold (2014) [11].

We observed high genetic diversity within both subspecies, resulting in a large number of different haplotypes, but these haplotypes often differ only in single substitutions between haplotypes (S1 and S2 Figs). The occurrence of several exclusive haplotypes at different localities suggest large population sizes as mentioned for species of *Poecilimon* [36]. This factor is known to result in a long persistence of ancestral haplotypes in a population [37,38].

Low dispersal capacities resulting in strongly subdivided populations and high population densities, could explain why most of the genetic variation is observed between individuals within the sampling sites (for both subspecies; up to 70%) as calculated by the AMOVA (S6 and S7 Files). Nevertheless, we could also show that nearly 20% of the genetic variation in the dataset is explainable by our new subspecies affiliation, based on the Barrier results.

We could identify IBD as a mechanism generating structure within the species as the geographical coordinates significantly explained 15.6% of the genetic variability (based on the ITS dataset). When analyzed within subspecies another big part of the genetic variation (14.9% for Pvv and 12.8% for Pvm) was explained by the coordinates (Table 3), which means that on a smaller geographic scale IBD is as important as on the entire distribution range.

Based on laboratory experiments we expect gene flow between the two subspecies of *P*. *veluchianus* to be reduced. Reinhold (1994) [10] found a reduced number of sperm for F1-hybrid males with a *P*. *v*. *veluchianus* mother. Therefore, we expect different amounts of shared haplotypes between the subspecies for both subspecies. Instead both subspecies feature a similar amount of haplotypes shared between subspecies (Pvv = 27.59%; Pvm = 23.53%; χ^2^-test: χ^2^ = 0.27, df = 1, p > 0.6). When locations close to the new contact zone were considered, only two haplotypes are shared across the contact zone.

As for *P*. *veluchianus*, males are the sound producing sex and waiting for females to approach them for mating [39], we would expect females to disperse on a wider range than males. Surprisingly the result of the dbRDA did not show an influence of sex on the genetic pattern (Table 3), but when we tested for IBD for both sexes separately, in females 19%, whereas in males only 10% of the variability was explained by the coordinates. Hence, the phenomenon of IBD was stronger in females than in males. This indicates that despite the necessity for females to walk around to locate a mating partner, males seem to disperse on a wider geographic scale, possibly to find an optimal spot to attract females.

To investigate whether the DNA-sequences show signs of non-random effects or evolve neutrally we calculated Fu’s and Li’s F* and D* for *P*. *veluchianus*. As both values were significant for the species, we concluded the ITS sequences evolved non-randomly (Table 4). Negative values indicate natural selection or the experience of a recent bottleneck with a subsequent phase of range expansion acting on the species [32]. Furthermore, this suggests that an excess of mutations in the recent past is more likely than in the more distant past [40]. On the subspecies level this effect is present in both subspecies but significant only for *P*. *v*. *minor*.

The findings based on the CR marker are in contrast to the results based on the ITS marker. The only barrier to gene flow determined by Barrier based on the CR marker is separating the two sampling sites in the most western part of the distribution range, which supports IBD. For the remaining distribution range of *P*. *veluchianus* the picture displayed is rather unstructured. Discordance between nuclear markers and a mitochondrial one has been observed in several biogeographic studies and might be caused by several different reasons like introgression, incomplete lineage sorting, disparity in range size or in abundance between hybridizing groups (reviewed in [41]). Responsible factors for the observed discrepancy and whether this might be due to saturation of the mitochondrial marker cannot be finally answered. But as we found evidence for a hybrid zone between both subspecies, the introgression of mitochondrial haplotypes from one subspecies into the other could be one possible explanation [42] and thus the results based on the nuclear marker seems to be more reliable.

The frequency of haplotypes which are shared between the two subspecies along the new contact zone is quite high and most prominent at a transect (see Fig 5 localities 04, 08, 09, 10, 14, 17, 20 and 21). This might indicate the occurrence of hybrids at these localities and it still remains to be tested how frequent they are. Individuals of both subspecies are rather hard to distinguish in the field as they are morphologically similar, besides for body size. As this feature is strongly influenced by the size of the mother, the body size of hybrids depends on the mothers’ subspecies identity [10]. But the actual morphological characteristics of hybrids are quite hard to estimate, as the body size differences between the subspecies expected to disappear in the hybrid zone. Based on laboratory experiments we know hybrids with a *P*. *v*. *veluchianus* mother grow bigger than pure *P*. *v*. *minor* individuals and hybrids with a *P*. *v*. *minor* mother stay smaller than pure *P*. *v*. *veluchianus* individuals [10]. Hence the size differences between both subspecies should be smaller in the hybrid zone if the gene flow between both subspecies is similar. Instead the size differences between both subspecies do not differ drastically within and outside the contact zone but tend to be larger in the contact zone.

Nevertheless, we found genetic support for the two subspecies of *P*. *veluchianus* and therefore corroborate the findings, based on the investigation of two adjacent sample sites, of Heller & Reinhold (1993) [9]. We hypothesize speciation in progress for this subspecies, as strong prezygotic isolation mechanisms are missing between the two parapatricaly distributed subspecies [9]. Whether this is a case of parapatric speciation, with the use of different niches or due to sexual selection and assortative mating [3,15,43–45], a secondary contact after an allopatric phase [2,46], the consequence of historical demographic processes, or the studied subspecies are part of a more widely distributed ring species [47] is still unknown. Ring species are an accepted way of how speciation with gene flow can occur. It has been shown that IBD has an important influence on the population structure and the parapatric distribution of ring species [48,49]. Therefore it is possible that *P*. *veluchianus* is part of a ring species. To further investigate this hypothesis, samples of closely related species of *Poecilimon* are necessary. Our findings are also able to be interpreted with non-ecological speciation were no geographical barrier is needed, but sexual selection plays a major role. This way of speciation is consistent with the neutral biodiversity theory (NBT), based on Hubbel (2001) [50] (reviewed in [51]). It is based on variances in population densities and restricted dispersal abilities, though drift and neutral selection are also considered as important forces for speciation. In our data we found evidence for these forces acting on the population structure of *P*. *veluchianus* (Table 4), also the findings of Reinhold (1994) [10] are consistent with NBT. As both subspecies are distributed in different altitudes with *P*. *v*. *veluchianus* occurring above 380 m and *P*. *v*. *minor* occurring also below this altitude and the lack of a strong prezygotic barrier this probably supports a secondary contact zone. The missing premating barrier suggests a rather weak selection against hybrids [52] and might also indicate speciation in progress.

An extension of the former mate-choice tests and the cross-mating-experiments [10,13] according to the number of sample sites tested is necessary to clarify whether subspecies differences in the examined behaviors are real differences and not only mirroring variation or showing a pattern of character displacement. Also AFLP’s and/or microsatellite data could add further insight to the question of ongoing speciation in *P*. *veluchianus* and the more detailed characterization of the suggested hybrid zone of the two subspecies.

## Conclusion

The population genetic analysis, based on the nuclear ITS marker revealed one main barrier to gene flow within the range of *Poecilimon veluchianus*, and thus supports a possible hybrid or contact zone between the two subspecies. The existence of hybrids cannot be finally clarified but might be indicated by the high frequencies of shared haplotypes between both subspecies which are quite prominent along the contact zone. In contrast the mitochondrial CR marker does not clearly support the separation of *P*. *veluchianus* into two subspecies with restricted gene flow and a clear contact zone. The factors responsible for the observed discrepancy between both markers, potentially be due to saturation of the mitochondrial marker cannot be finally answered. In general IBD has a great influence on the observed genetic pattern with a more prominent influence in females than in males indicating a sex biased dispersal capacity. Both subspecies show signs of non-random evolution, which either could indicate natural selection or that the species experienced a recent bottleneck and is now in a phase of range expansion. Testing both subspecies separately for departure of neutral evolution, the results are only significant for *P*. *v*. *minor*. This might indicate that *P*. *v*. *minor* is either more affected or that only this subspecies experienced the bottleneck. The frequency of haplotypes which are shared between the two subspecies along the contact zone is quite high but surprisingly a difference in shared haplotypes between both subspecies, as expected due to the quasi sterility of F1-hybrid-males with a *P*. *v*. *veluchianus* mother, was not observed. Together with our findings, the previously published lack of strong prezygotic barriers combined with the presence of postzygotic mating barriers suggest that a secondary contact after an allopatric phase is rather likely for the two subspecies of *P*. *veluchianus*.

## Supporting Information

S1 Fig Average number of pairwise differences of the CR marker. Between population p-distances are coloured in green, in the upper left side of the matrix. Within population p-distances coloured in orange, diagonal line of matrix. The net number of nucleotide differences between pairs of populations are coloured in blue, in the lower right side of the matrix.
(TIF)

S2 Fig Average number of pairwise differences of the ITS marker. Between population p-distances are coloured in green, in the upper left side of the matrix. Within population p-distances coloured in orange, diagonal line of matrix. The net number of nucleotide differences between pairs of populations are coloured in blue, in the lower right side of the matrix.
(TIF)

S1 File Improved Alignment of the CR marker. (FAS)

S2 File Improved Alignment of the ITS marker. (FAS)

S3 File Number of shared and exclusive haplotypes of sample sites for the CR marker, according to the hypothesized contact zone. (XLS)

S4 File Number of shared and exclusive haplotypes of sample sites for the ITS marker, according to the hypothesized contact zone. (XLS)

S5 File Number of shared and exclusive haplotypes of sample sites for the ITS marker, according to the new contact zone calculated by Barrier. (XLS)

S6 File AMOVA results for the ITS marker. Partitioning of sample sites to subspecies along the hypothesized contact zone. (XLS)

S7 File AMOVA results for the ITS marker. Partitioning of sample sites into subspecies along the calculated barrier 1 for the ITS marker. (XLS)
