# Supplementary figures and images for: Speciation Progress: A Case Study on the Bushcricket Poecilimon veluchianus
Source: PLoS One. 2015 Oct 5;10(10):e0139494. doi: 10.1371/journal.pone.0139494 (PMC4593647; doi:10.1371/journal.pone.0139494)

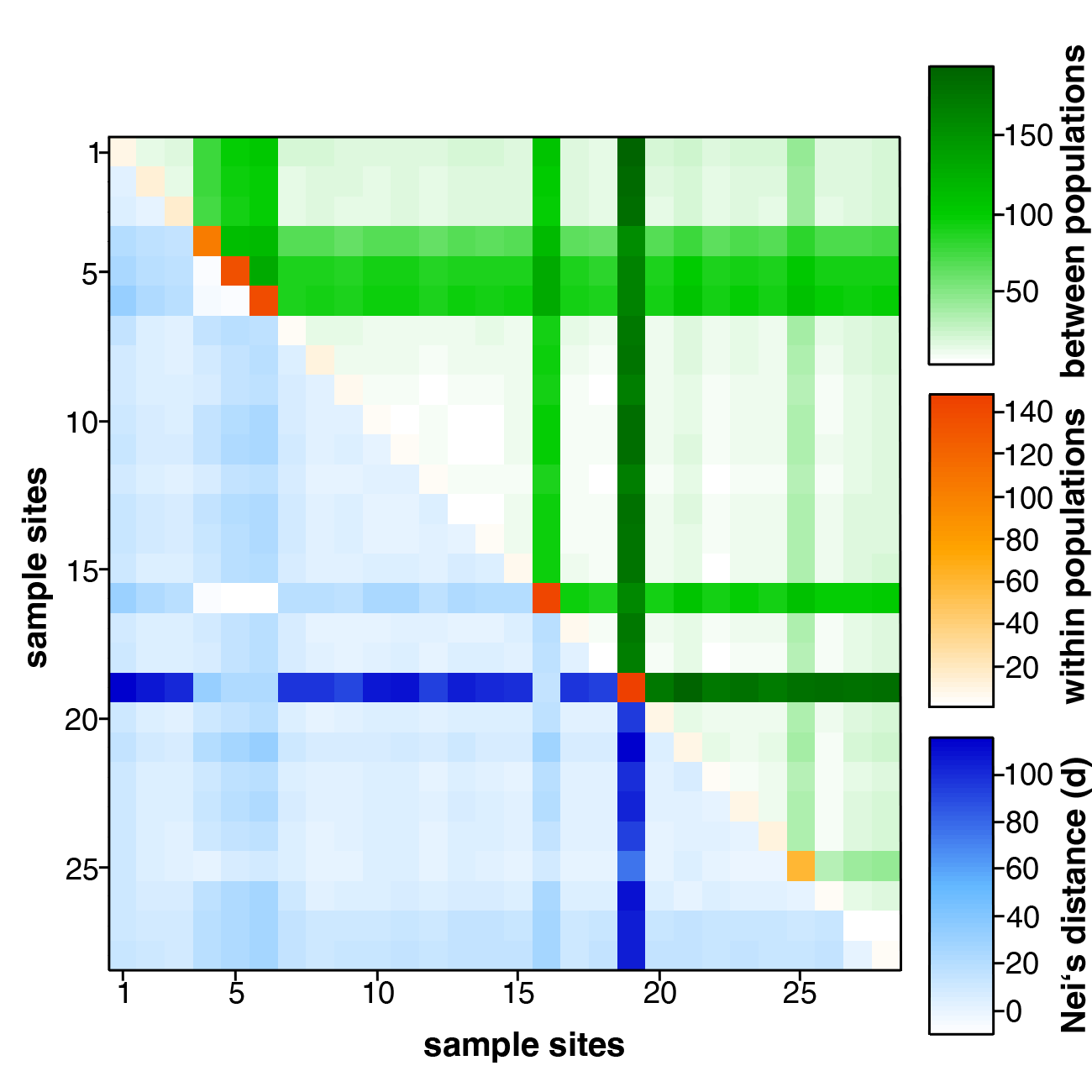

Supplement: S1 Fig — Between population p-distances are coloured in green, in the upper left side of the matrix. Within population p-distances coloured in orange, diagonal line of matrix. The net number of nucleotide differences between pairs of populations are coloured in blue, in the lower right side of the matrix. (TIF) [file pone.0139494.s001.tif]

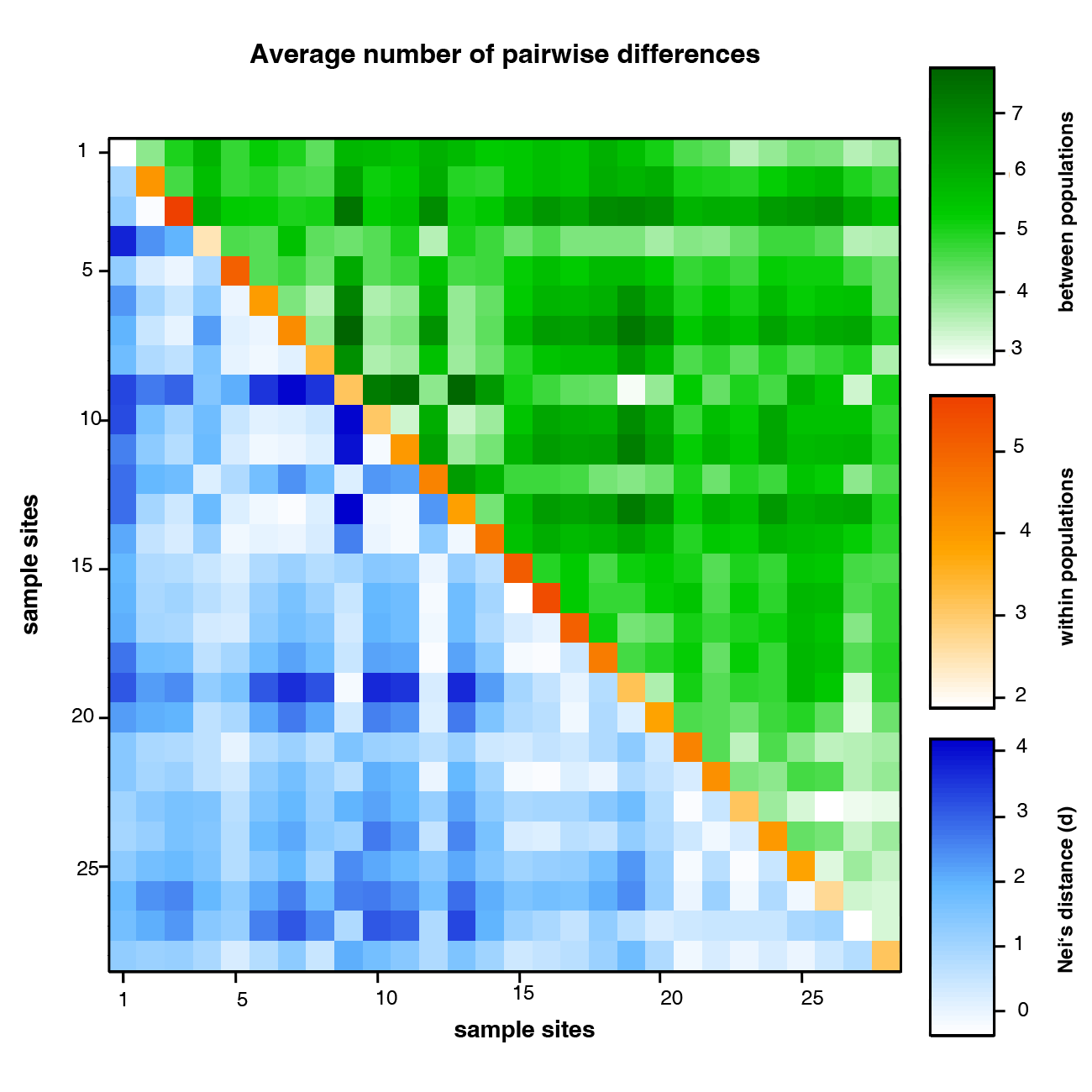

Supplement: S2 Fig — Between population p-distances are coloured in green, in the upper left side of the matrix. Within population p-distances coloured in orange, diagonal line of matrix. The net number of nucleotide differences between pairs of populations are coloured in blue, in the lower right side of the matrix. (TIF) [file pone.0139494.s002.tif]
